# Reversible Mechano-Regulation of Cellular Senescence: Effects of Substrate Stiffness on Cells

**DOI:** 10.3390/cells15151380

**Published:** 2026-07-30

**Authors:** Jin Young An, Sung Won Jang, Yun Dong Goo, Ju Hwan Kim, Da Hong Kim, Su A Park, Majid Ebrahimi Warkiani, Jae Ho Lee

**Affiliations:** 1 Department of Biomedical Science, College of Life Science, CHA University, Pocheon 11160, Republic of Korea; 2 Nano-Convergence Manufacturing Research Division, Korea Institute of Machinery and Materials (KIMM), Daejeon 34103, Republic of Korea; 3 School of Biomedical Engineering, University of Technology Sydney, Ultimo, NSW 2007, Australia; 4 CHA Fertility Center Seoul Station, Seoul 04637, Republic of Korea

**Keywords:** biomechanical cues, cellular senescence, reversibility, mechanobiology, aging

## Abstract

**Highlights:**

**What are the main findings?**

- Hydrogel stiffness regulates cellular morphology, senescence-associated phenotypes, and longevity-related gene expression.
- A soft mechanical microenvironment enhances autophagy and partially reverses aging-associated cellular characteristics.

**Abstract:**

Aging involves the accumulation of molecular alterations within cells and the extracellular matrix, resulting in cellular senescence and declining physiological functions. This study investigates the correlation between the biophysical environment and cellular aging, specifically examining how mechanical and biochemical cues affect cellular senescence and tissue degeneration. Cells were cultured on acrylamide hydrogels of different stiffnesses (4 and 19 kPa), and their mechanical properties were characterized by measuring Young’s modulus via compression tests. Cell proliferation, morphology, gene and protein expression, and autophagy activity were assessed using multiple assays and imaging techniques. Cells cultured on stiff hydrogels exhibited elongated morphologies, whereas cells on soft hydrogels formed spherical clusters. Notably, longevity-associated genes were upregulated in cells cultured on softer substrates. Reversibility experiments demonstrated that the aging phenotype could be reversed by modulating mechanical culture conditions, with softer environments enhancing autophagic activity. In summary, hydrogel stiffness significantly impacts aging-related cellular behavior. These findings suggest biomechanical cues as a promising strategy to promote cellular rejuvenation and combat aging.

## 1. Introduction

Aging is a multifactorial process characterized by the progressive accumulation of molecular alterations within cells and the extracellular matrix (ECM). These changes ultimately lead to cellular senescence, a state of permanent cell cycle arrest, and the gradual decline in physiological functions [1,2]. Recent studies have emphasized that the mechanical properties of the ECM, particularly its stiffness, serve as critical regulators of cellular homeostasis. In many tissues, aging is accompanied by significant alterations in the hierarchical organization of collagen and proteoglycans, leading to increased ECM stiffness [3]. Such changes in the physical microenvironment are now recognized as potent factors that can modulate biological behaviors, including proliferation, gene expression, and the onset of aging-related phenotypes [4].

Mechanical cues from the cellular microenvironment are key regulators of cell fate and function through mechanotransduction. Cells dynamically interact with their surroundings, sensing mechanical cues like substrate stiffness to guide internal signaling pathways [5,6]. While much research has focused on how stiffness influences development or stem cell differentiation, its direct role in driving or mitigating cellular senescence in somatic cells remains less explored. In particular, the dysregulation of mechanobiological processes in stiffened aged tissues has been linked to impairments in cardiovascular and musculoskeletal systems [7]. However, whether these mechanical signals are merely a consequence of aging or an active driver that can be manipulated to reverse senescent phenotypes is a critical question that requires rigorous validation.

Despite the growing understanding of mechanobiology, the precise molecular mechanisms by which environmental stiffness mediates cellular senescence are not fully understood. It remains unclear whether a stiffened microenvironment can directly accelerate the transition of healthy cells into a senescent state, and more importantly, whether restoring a compliant, “young” mechanical environment can confer rejuvenating effects or mitigate established senescence markers [8]. Addressing these questions is essential for developing targeted anti-aging interventions that go beyond biochemical signaling to include the modulation of physical factors such as extracellular stiffness and spatial constraints [9].

This study investigates how substrate stiffness dictates cellular senescence in HEK293 cells and whether these effects are reversible. To establish a highly standardized and genetically homogenous platform for dissecting fundamental mechanotransduction pathways without confounding cellular variables, HEK293 cells were utilized as a model system. Specifically, we aimed to determine whether a stiffened microenvironment actively drives senescence-associated phenotypes and, conversely, whether transitioning cells to a compliant environment can physically rescue or mitigate these aging markers. By focusing on the dynamic reversibility of cellular aging, this work seeks to provide broader mechanistic insights into how biophysical cues fundamentally regulate cellular fate and homeostasis.

## 2. Materials and Methods

### 2.1. Preparation of Cells

We purchased HEK293 (Human Embryonic Kidney: 293 [HEK-293], Catalog No. KCLB No. 21573) cells from the Korea Cell Line Bank (KCLB). The cell line was obtained in March 2024. The identity of the HEK293 cell line (RRID: CVCL_0045) was confirmed by short tandem repeat (STR) profiling, and the results matched the reference STR profile for HEK293, thereby confirming the authenticity of the cell line. The cells were maintained in HyClone media (DMEM with High Glucose) containing 10% fetal bovine serum and 1% Penicillin–Streptomycin (10,000 U/ML). Cells were incubated at 37 °C in a humidified incubator atmosphere with 5% CO_2_. Cells were cultured in DMEM (Hyclone, Logan, UT, USA) supplemented with 10% fetal bovine serum (Gibco, Carlsbad, CA, USA) and 1% penicillin/streptomycin (Gibco, Carlsbad, CA, USA). A total of 1 × 10^5^ cells were seeded on a cell culture dish (90 × 20 mm) (SPL, Anseong, Gyeonggi, Republic of Korea) and kept in an incubator containing 5% CO_2_ at 37 °C. Cells were passaged every 4 days when the cells reached 70–80% confluency. Cells were collected after expansion at passages 12–13.

### 2.2. Preparation of 4 and 19 kPa by Polyacrylamide Hydrogel

To prepare hydrogels with distinct mechanical properties, two formulations were used. The soft hydrogel consisted of 1.25 mL of 40% acrylamide, 0.75 mL of 2% bis-acrylamide, and 7.89 mL of distilled water, while the stiff hydrogel was composed of 2 mL of 40% acrylamide, 1.32 mL of 2% bis-acrylamide, and 6.57 mL of distilled water. To each formulation, 100 µL of 10% ammonium persulfate (APS), 10 µL of TEMED, and 10 µL of penicillin–streptomycin (10,000 U/mL) were added to reach a final volume of 10 mL (Table S1). A total of 200 µL of each mixture was poured onto a parafilm-covered glass plate and overlaid with an 18 mm cover glass. After polymerization (~30 min), the cover glass was inverted to expose the hydrogel surface, which was activated with 5 mM Sulfo-SANPAH under UV light for 5 min, washed with 1× PBS, and incubated overnight at 4 °C. The gels were then coated with 60 µL of collagen type I and incubated overnight at 4 °C. Before cell seeding, the hydrogels were washed three times with 1× PBS and soaked in culture media for 4–5 h at 37 °C. HEK293 cells were seeded at a density of 5 × 10^4^ cells per well in 12-well plates and cultured on the hydrogels for 4 days.

### 2.3. Mechanical Properties

The mechanical properties of the samples were evaluated by determining the Young’s modulus through a compression test using a universal testing machine (UTM). Cylindrical samples with dimensions of 4 mm in diameter and 2 mm in height were prepared and incubated in 1× PBS at 37 °C for 24 h to maintain consistent conditions. The compression test was conducted using a 20 kgf load cell at a constant crosshead speed of 1 mm/min. To ensure statistical robustness and reproducibility, twelve independent hydrogel specimens (*n* = 12) were measured for each experimental group. The Young’s modulus was calculated from the linear slope of the initial elastic region of the stress–strain curves.

### 2.4. Cell Proliferation Assay Using CCK

The proliferation of HEK293 cells cultured on 2D hydrogels was evaluated using the WST-8 Cell Viability Assay Kit (QM2500; BioMax, Seoul, Republic of Korea), a colorimetric method for measuring cell proliferation and cytotoxicity. HEK293 cells (5 × 10^4^) were cultured on hydrogels with stiffness values of 4 kPa and 19 kPa. To prevent interference from cells that may have adhered to the bottom of the original culture plate, the hydrogel constructs along with their culture medium were carefully transferred to new 12-well plates prior to the assay. WST-8 reagent was added at 1/10 the volume of the culture medium, and the mixture was incubated for 90 min at 37 °C. Absorbance at 450 nm was measured using a microplate spectrophotometer (Epoch; BioTek™ Microplate Spectrophotometer, Winooski, VT, USA). The cell proliferation was evaluated at 0, 2, and 4 days after seeding by measuring WST-8 absorbance at each time point.

### 2.5. Fluorescence Imaging of Phalloidin-Stained Cells

A total of 5 × 10^4^ cells were seeded in a 12-well plate (SPL, Korea). After 24 h, cells were fixed using 4% PFA and then permeabilized with 1% Triton X solution. F-actin structure and Hoechst 33342, which labels nuclei, were treated for 20 min and 5 min, respectively. Stained cells were imaged using an inverted fluorescence microscope equipped with a DS-5i camera (Eclipse Ti-Ul; Nikon, Tokyo, Japan).

### 2.6. Senescence-Associated (SA) β-Galactosidase Assay

Senescence-associated β-galactosidase (SA-β-Gal) activity was assessed using an X-gal–based Senescence β-Galactosidase Staining Kit (ab65351; Abcam, Cambridge, UK) according to the manufacturer’s protocol. After fixation, cells were incubated with the staining solution at 37 °C in a CO_2_-free environment overnight. The appearance of a blue precipitate was used to identify SA-β-Gal-positive cells. The extent of senescence was quantified by threshold analysis using ImageJ software (version 1.54t), and the percentage of SA-β-Gal-positive area was calculated relative to the total image area.

### 2.7. Gene Expression Analysis by Reverse Transcription PCR

Total RNA was extracted from harvested cells using the easy-Spin™ Total RNA Kit (iNtRON, Seongnam, Republic of Korea), and RNA concentration was measured using a microplate spectrophotometer (Epoch™ Microplate Spectrophotometer, BioTek, Winooski, VT, USA). The RNA was then diluted to a final concentration of 100 ng/µL. Reverse transcription was performed using RT PreMix (dT20) (Bioneer, Republic of Korea) on a SimpliAmp™ Thermal Cycler (Life Technologies, Carlsbad, CA, USA) to synthesize complementary DNA (cDNA). For PCR amplification, a 20 µL reaction was prepared using AccuPower^®^ Taq PCR Premix (K-2602, Bioneer, Daejeon, Republic of Korea), containing 18 µL of premix with primers and 2 µL of cDNA (100 ng). PCR was carried out for 32 cycles, including an initial denaturation step at 95 °C for 1 min, followed by denaturation at 95 °C for 30 s, annealing at 60 °C for 30 s, and extension at 72 °C for 50 s. PCR products were separated on a 1.5% agarose gel, and band intensities were quantified using ImageJ software (version 1.54t).

### 2.8. Protein Expression Analysis by Western Blotting

The protein in the sample was extracted using PRO-PREPTM Protein Extraction Solution (17081, INtRON, Seongnam, Republic of Korea) and quantified using Protein Quantification Kit (BSA Standard, BCA0500, Biomax, Seoul, Republic of Korea). Samples were then boiled in 4X Laemmli sample buffer (1610747, Bio-Rad, Hercules, CA, USA) containing 2-mercaptoethanol (1610710, Bio-Rad, Hercules, CA, USA). The same amount of protein was loaded into each well of an 8% sodium dodecyl sulfate polyacrylamide gel. The gel was electrophoresed at 0.02 A for 30 min and then at 0.04 A for 2 h. The proteins were transblotted onto a nitrocellulose membrane (BR162-0112, Bio-Rad, Hercules, CA, USA) at 100 V for 80 min. The membrane was incubated with blocking buffer (TBS-T containing 5% bovine serum albumin) for 1 h and then with a primary antibody solution overnight at 4 °C. The primary antibodies used were mouse anti-beta Actin (MA5-15739, Thermo Fisher Scientific, Waltham, MA, USA), mouse anti-SIRT1 (sc-74465, Santa Cruz Biotechnology, INC, Dallas, TX, USA), rabbit anti-pAMPK (701068, Waltham, MA, Thermo Fisher Scientific), mouse anti-AMPK (AHO1332, Thermo Fisher, Waltham, MA, USA), rabbit anti-pmTOR (Ser2448, Cell Signaling, Danvers, MA, USA), rabbit anti-mTOR (2983T; Cell Signaling, Massachusetts), rabbit anti-pAKT (44621-G, Thermo Fisher, Waltham, MA, USA), and rabbit anti-AKT (MA5-14916, Invitrogen, Carlsbad, CA, USA). The blotted membrane was washed with TBS-T and incubated with horseradish peroxidase-conjugated anti-mouse (G-21040, Invitrogen, Carlsbad, CA, USA) and anti-rabbit (31460, Invitrogen, Carlsbad, CA, USA) secondary antibodies for 60 min at room temperature. Immunoreactive bands were detected using the enhanced Miracle-StarTM (Western Blot Detection System, 16028, INtRON, Seongnam, Republic of Korea). Images of the bands were acquired using ImageSaver version 6 (ATTO Corporation, Taito-ku, Tokyo, Japan). The experiment was repeated three times under the same conditions with different samples.

### 2.9. Autophagy Assay

To investigate the autophagy activity of cells on the hydrogel, they were stained with an autophagy assay kit (MAK138, Sigma-Aldrich, St. Louis, MO, USA), following the manufacturer’s protocol. HEK293 cells were cultured on 4 kPa and 19 kPa hydrogel in a regular DMEM medium. The hydrogel was transferred to a different 12-well plate, and the cells attached to the bottom of the plate were removed. The medium was removed from the cells, and 1 mL of the autophagosome detection reagent working solution was added to each well. The cells were incubated at 37 °C with 5% CO_2_ for 1 h. After incubation with stain solution, the cells were washed twice with 1 mL of wash buffer in each well. Autophagy activity in the cells was imaged using an inverted fluorescence microscope (Eclipse Ti2-E, NIKON, Tokyo, Japan) equipped with a DS-5i camera. Fluorescence intensities in the fluorescence microscope images were quantified using Image J.

### 2.10. Statistical Analysis

All data are expressed as mean ± SEM. Experimental results were obtained from at least three independent experiments (*n* ≥ 3), and the specific sample size (n) for each analysis is indicated in the corresponding figure legends. Prior to statistical testing, data distribution and variance were evaluated to satisfy the assumptions of the analysis. Statistical significance between groups was determined using two-sided one-way analysis of variance (ANOVA), followed by the Holm–Sidak post hoc test for multiple comparisons. All statistical analyses and data visualizations were performed using SigmaPlot version 12.5 software. Statistical significance was defined as * *p* < 0.05, ** *p* < 0.01, and *** *p* < 0.001.

## 3. Results

### 3.1. The Mechanical Property Analysis of Polyacrylamide Hydrogels

**Figure 1:**
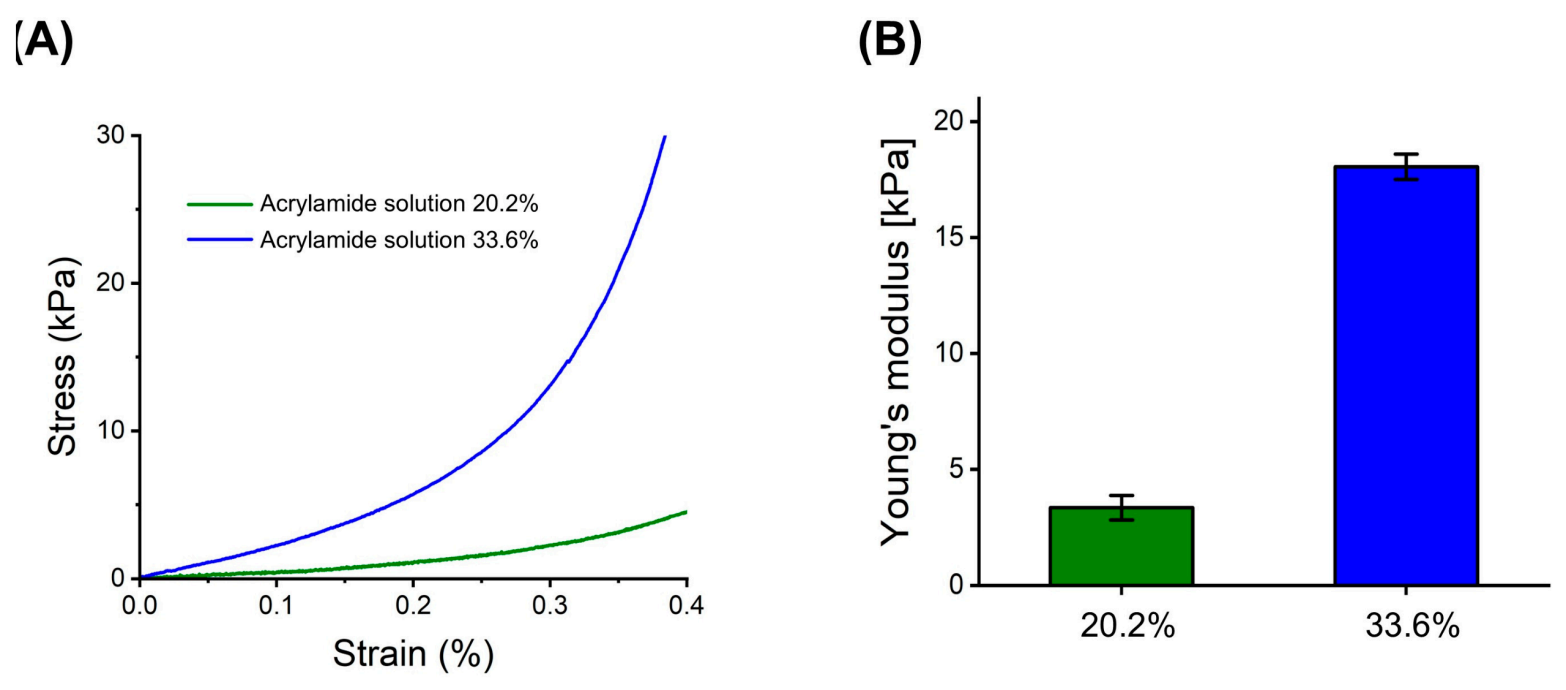
**Mechanical characterization of hydrogels with different acrylamide concentrations.** (**A**) Stress–strain curves of hydrogels prepared with 20.2% and 33.6% acrylamide solutions. The hydrogel with a lower acrylamide concentration (20.2%) exhibits reduced stress generation under strain, reflecting its softer and more compliant mechanical nature. In contrast, the 33.6% hydrogel shows markedly higher stress with increasing strain, consistent with enhanced stiffness. (**B**) Quantification of Young’s modulus derived from the stress–strain curves. The 20.2% hydrogel demonstrates an average modulus of approximately 4 kPa, while the 33.6% hydrogel reaches 19 kPa. These measurements highlight the tunability of hydrogel stiffness as a function of acrylamide concentration, enabling the generation of soft versus stiff microenvironments. Data are presented as mean ± SEM from *n* = 12 independent hydrogel samples per group. Statistical comparisons were not performed as these values represent intrinsic material characterization.

To compare the formation of relatively soft and stiff hydrogels, polyacrylamide hydrogels with varying concentrations were cultured and analyzed. The compressive modulus increased proportionally with the bis-acrylamide concentration. The Young’s modulus was measured as 3.35 ± 0.529 kPa for 20.2% polyacrylamide hydrogels and 18.06 ± 0.545 kPa for 33.6% polyacrylamide hydrogels (Figure 1).

### 3.2. Substrate Stiffness-Dependent Modulation of Cell Morphology and Proliferation

**Figure 2:**
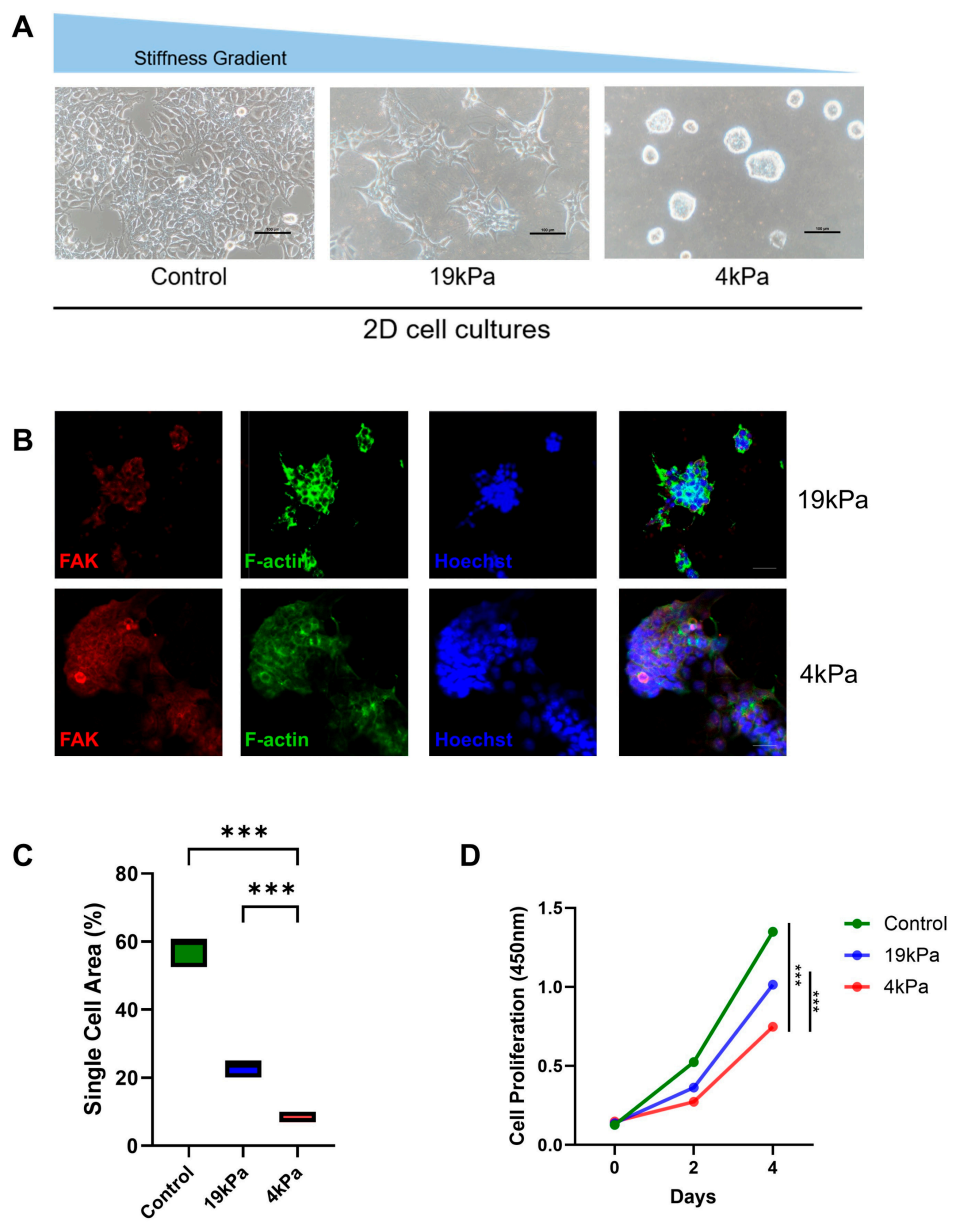
**The cell proliferation and morphology of HEK293 in biomaterial hydrogels.** (**A**) Representative morphology of HEK293 cells cultured on a culture-treated polystyrene dish (control) and in 2D biomaterial hydrogels at day 4 of culture. (**B**) Confocal images of HEK293 cells on the 4 kPa and 19 kPa hydrogels show fluorescence co-immunocytochemistry staining with anti-FAK polyclonal antibody (red), F-actin (green), and Hoechst (blue) for nuclear staining. (**C**) The cell area (%) of HEK293 spread on the hydrogel surface after 4 days. (**D**) Cell proliferation (5 × 10^3^ cells). The absorbance at 450 nm was measured at 90 min. Data are presented as mean ± SEM (*n* = 3 independent experiments). Statistical significance was determined by one-way ANOVA followed by the Holm–Sidak post hoc test (*** *p* < 0.001).

Substrate stiffness fundamentally altered the physical and biological characteristics of HEK293 cells. As shown in Figure 2A, cells cultured on stiff hydrogel (19 kPa) exhibited a distinct fibroblast-like morphology, characterized by an elongated, spindle-shaped body and prominent cellular branching. In stark contrast, cells on the soft hydrogel (4 kPa) failed to spread, instead forming compact, rounded clusters with minimal substrate interaction. To further examine cytoskeletal organization, we performed immunofluorescence staining for F-actin (Figure 2B). Cells on the soft hydrogel displayed circular, cortical F-actin structures consistent with their rounded morphology, whereas those on the stiff hydrogel showed elongated actin filaments aligned with their stretched morphology. To quantify these morphological differences, we measured the single-cell area under each condition. As shown in Figure 2C, cell spreading increased significantly with matrix stiffness, with the control group showing the largest average area. As shown in Figure 2D, proliferation was highest in the control group, moderate in the 19 kPa condition, and lowest in the 4 kPa condition.

### 3.3. The Mechanical Properties Induced Senescence-Associated Beta-Galactosidase (SA-β-X-Gal) Activity

**Figure 3:**
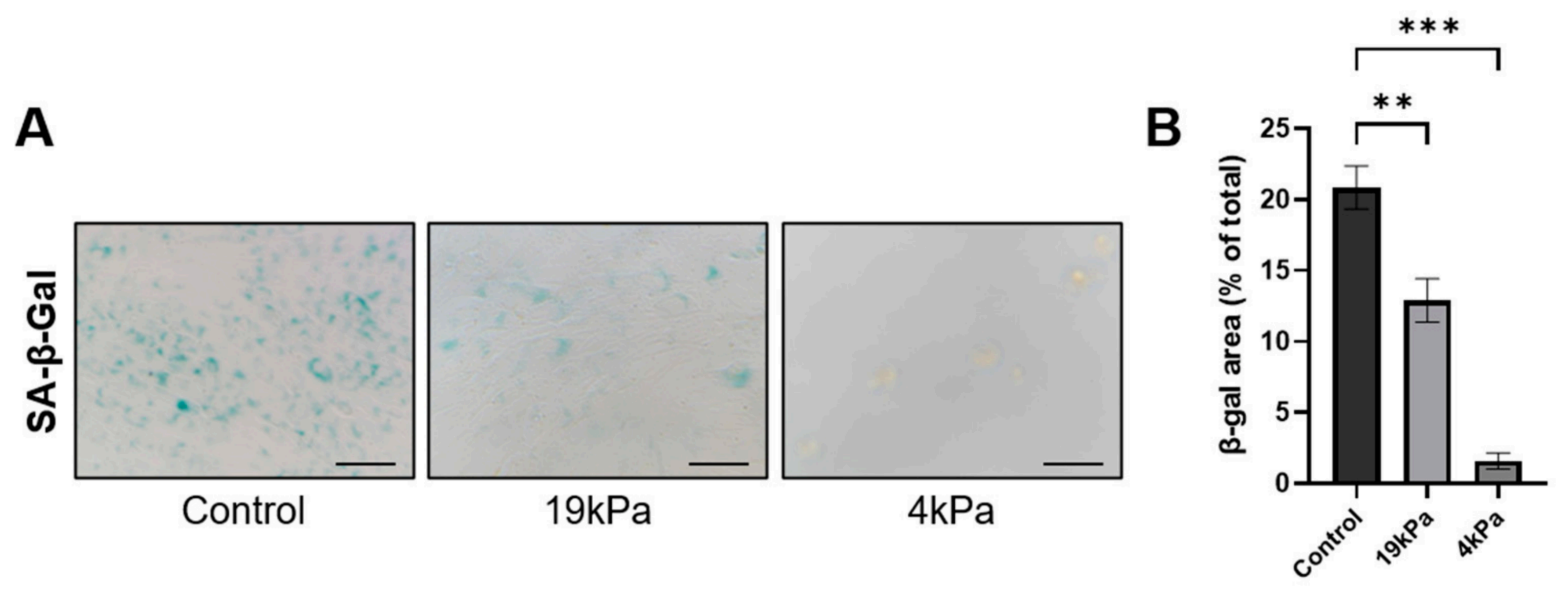
**X-gal staining of senescent HEK293 cells according to hydrogel stiffness.** (**A**) SA-β-Gal (senescence-associated β-galactosidase) activity was evaluated in HEK293 cells maintained on hydrogels with different mechanical properties (control, 19 kPa, and 4 kPa). (**B**) The extent of senescence was quantified as the percentage of SA-β-Gal-positive area relative to the total image area using threshold analysis. Data are presented as mean ± SEM (*n* = 5 independent experiments). Statistical significance was determined by one-way ANOVA followed by the Holm–Sidak post hoc test (** *p* < 0.01, *** *p* < 0.001).

Representative images of SA-β-Gal staining in cells cultured on substrates of varying stiffness are shown: Control, 19 kPa, and 4 kPa (Figure 3A). Cells on softer hydrogel (4 kPa) exhibited markedly reduced SA-β-Gal staining compared to control and 19 kPa conditions. Quantification of β-galactosidase-positive area as a percentage of total area revealed a stiffness-dependent decrease in senescence: Control (20.85%), 19 kPa (12.89%), and 4 kPa (1.26%) (Figure 3B).

### 3.4. Longevity-Related Gene Expression

**Figure 4:**
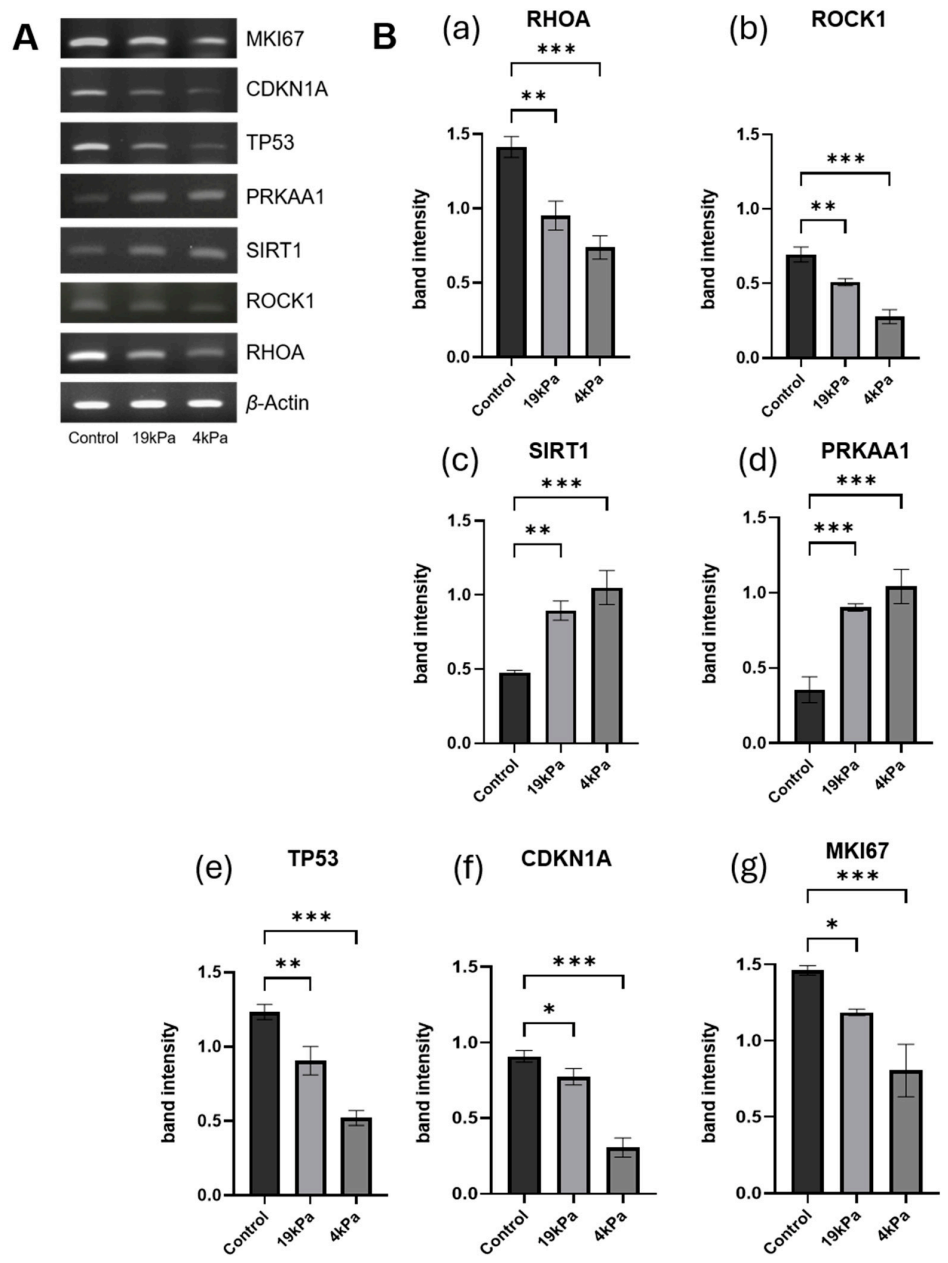
**Differential expressions of longevity-related genes assessed by RT-PCR.** (**A**) Representative RT-PCR gel electrophoresis images showing the expression of RhoA, ROCK1, SIRT1, PRKAA1, TP53, CDKN1A, and MKI67. (**B**) Relative gene expression levels of (**a**) RHOA, (**b**) ROCK1, (**c**) SIRT1, (**d**) PRKAA1, (**e**) TP53, (**f**) CDKN1A, and (**g**) MKI67 quantified by RT-PCR. Results are expressed as the mean ± SEM from three independent experiments (*n* = 3). Statistical analysis was performed using one-way ANOVA followed by the Holm–Sidak multiple comparisons test (* *p* < 0.05, ** *p* < 0.01, *** *p* < 0.001).

To determine whether key regulators of cellular aging respond differently to variations in biomechanical cues, we examined the expression of longevity-associated signaling pathways under soft versus stiff culture conditions. Figure 4A demonstrates that the expression levels of PRKAA1 and SIRT1 significantly increase under soft biomechanical conditions compared to both the stiff condition and the control group. Conversely, expression of mechanotransduction-associated effectors such as RhoA and ROCK1 is relatively downregulated under soft conditions compared to stiff substrates. Specifically, compared to the control group, cells in the soft (4 kPa) condition showed that (a) RhoA expression was reduced by 47.8%, and (b) ROCK1 expression by 60.2%. In contrast, (c) SIRT1 expression increased by 119.8%, and (d) PRKAA1 expression showed a 193.3% elevation. Furthermore, (e) TP53 and (f) CDKN1A expression levels decreased by 57.9% and 66.2%, respectively, while (g) MKI67 expression exhibited a 44.9% reduction (Figure 4B).

### 3.5. Longevity-Related Protein Expression

**Figure 5:**
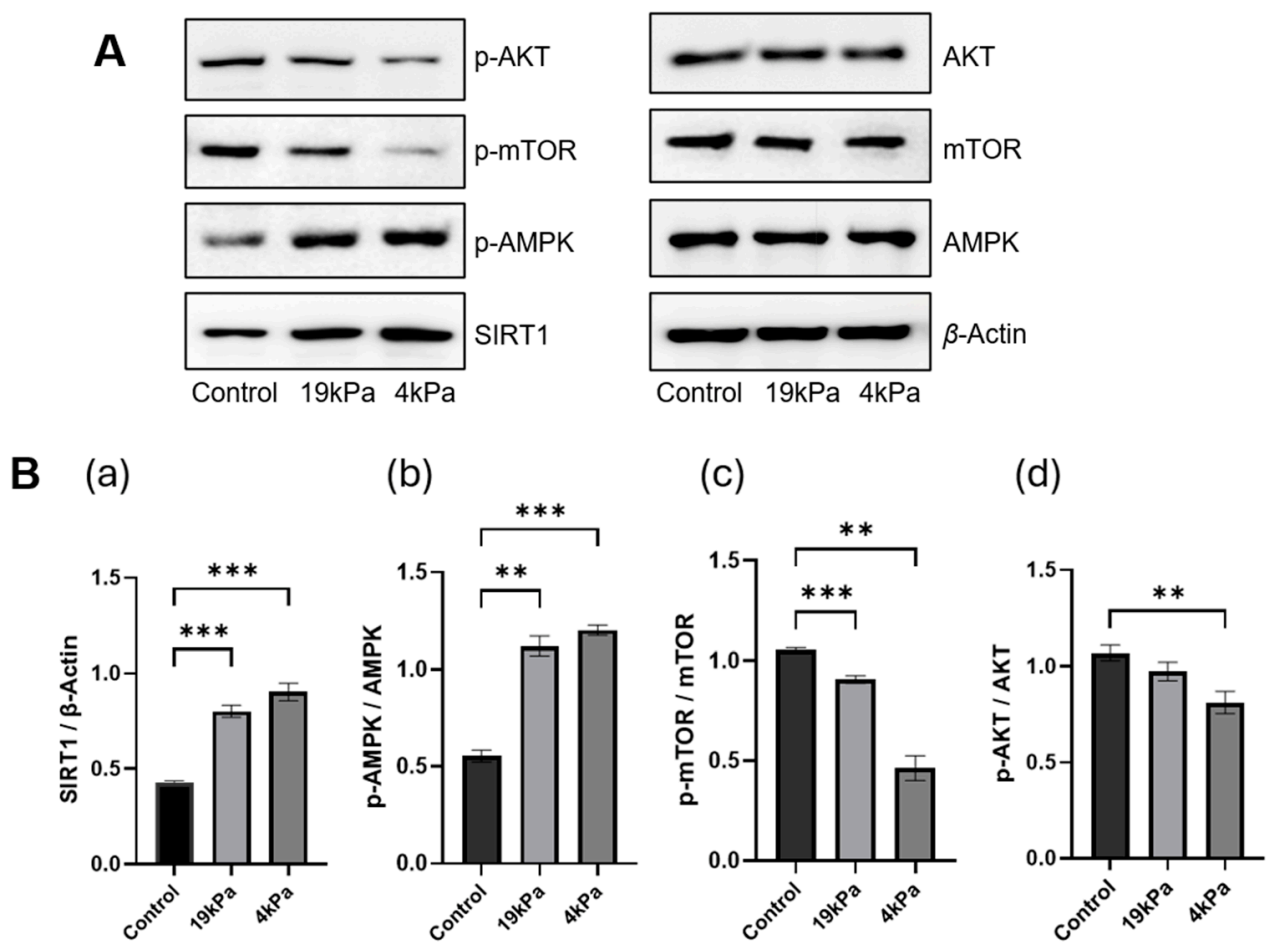
**Up- and down-regulation of longevity related protein analysis by Western blot.** (**A**) Representative Western blot images of SIRT1, AMPK, mTOR, and AKT. (**B**) Quantification of the relative protein expression levels of (**a**) SIRT1, (**b**) AMPK, (**c**) mTOR, and (**d**) AKT. Data are presented as mean ± SEM (*n* = 3 independent experiments). Statistical significance was determined by one-way ANOVA followed by the Holm–Sidak post hoc test (** *p* < 0.01, *** *p* < 0.001).

Figure 5 demonstrates that under soft biomechanical conditions, the expression levels of AMPK and SIRT1 are significantly increased compared to both stiff conditions and control. In contrast, the expression levels of mTOR and AKT are relatively decreased under soft culture conditions compared to stiff conditions (Figure 5A). Specifically, compared to the control group, cells cultured on the soft hydrogel (4 kPa) exhibited the following changes: (a) SIRT1 expression is increased by 111.6%, (b) AMPK expression is increased by 116.9%, (c) mTOR expression is decreased by 56.1%, and (d) AKT expression is decreased by 24.1%. These findings are consistent with prior studies showing that AMPK activation and mTOR suppression are hallmarks of longevity and metabolic reprogramming (Figure 5B).

### 3.6. Aging Recovery Study Between Soft and Hard Hydrogel Culture Conditions

**Figure 6:**
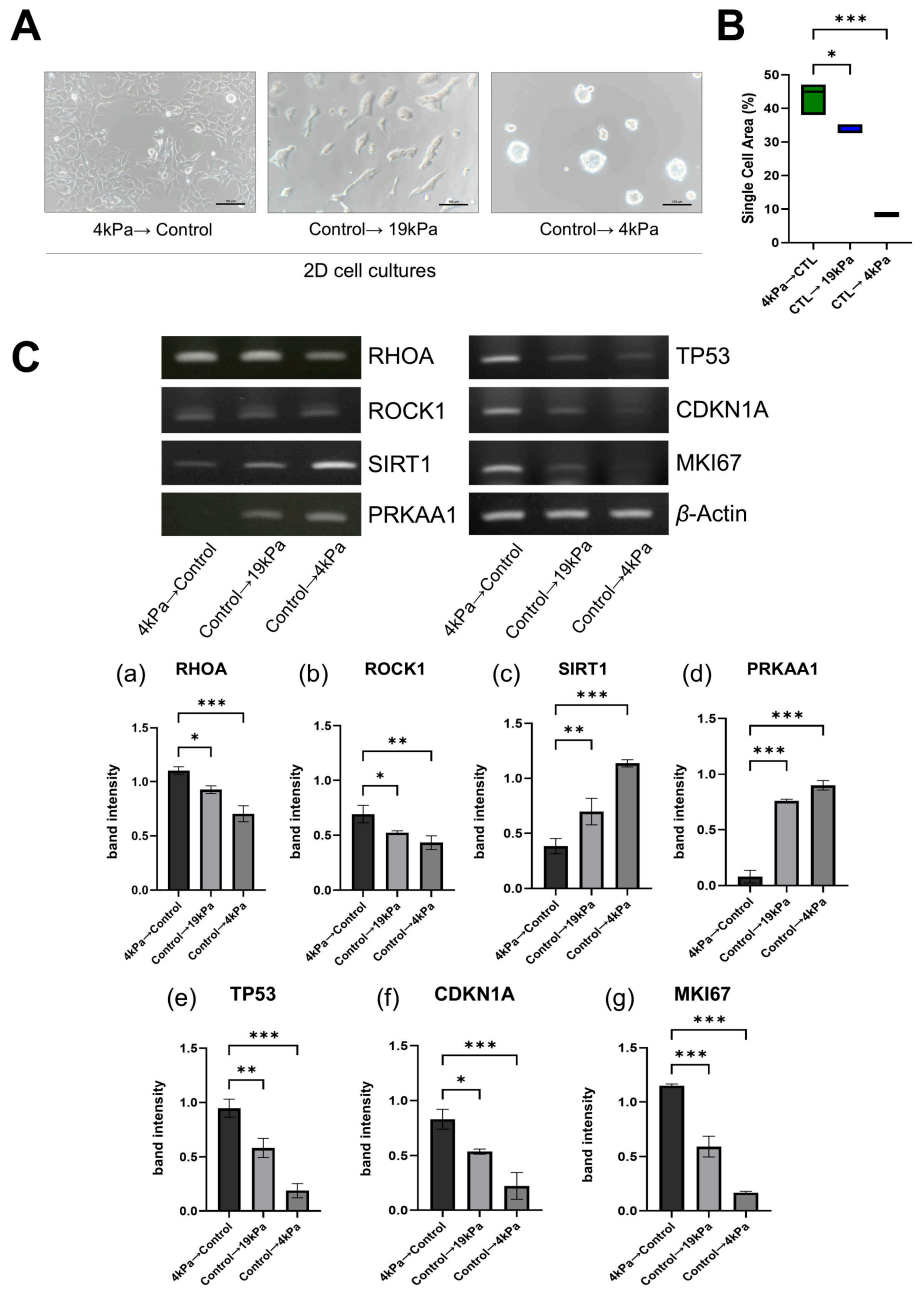
**Reversible cell behavior by altering the hydrogel environment.** (**A**) The proliferation and morphology of HEK293 cells in response to altered hydrogel environments. Cells grown in 4 kPa hydrogels were placed on Petri dishes, and cells grown in Petri dishes were cultured at 19 kPa and 4 kPa, respectively, to alter the mechanical environment. (**B**) The cell spreading area (%) of HEK293 on the modified hydrogel surfaces after 4 days. (**C**) Regulation in the expression of aging-associated genes, including (**a**) RhoA, (**b**) ROCK1, (**c**) SIRT1, (**d**) PRKAA1, (**e**) TP53, (**f**) CDKN1A, and (**g**) MKI67, were observed following changes in hydrogel stiffness. Results are presented as mean ± SEM obtained from three independent experiments (*n* = 3). Statistical significance among groups was assessed by one-way ANOVA followed by the Holm–Sidak post hoc test (* *p* < 0.05, ** *p* < 0.01, *** *p* < 0.001).

We investigated whether the aged phenotype induced by substrate stiffness is reversible when the mechanical culture environment is altered. We transferred cells between different mechanical conditions: from soft hydrogel to control (plastic), from control to stiff hydrogel (19 kPa), and from control to soft hydrogel (4 kPa). Remarkably, cells initially cultured on the 4 kPa hydrogel underwent a significant morphological transition upon transfer to the control substrate, exhibiting enhanced spreading and elongation (Figure 6A). This structural shift demonstrates that cellular phenotypes are not irreversibly fixed but remain dynamically responsive to changes in mechanical inputs.

Figure 6B quantifies changes in cell area and density following mechanical condition shifts. Cells moved from soft to stiff environments displayed increased spreading and larger cell area, while cells transferred from stiff to soft substrates exhibited reduced spreading and clustering. Figure 6C evaluates gene expression profiles after mechanical switching. Panels (a) and (b) show decreased expression of the mechanotransduction genes RhoA and ROCK1, and panels (e)–(g) demonstrate a reduction in the senescence and proliferation-associated markers TP53, CDKN1A, and MKI67. In contrast, panels (c) and (d) reveal increased expression of the longevity-associated genes SIRT1 and AMPK under soft culture conditions, indicating a shift toward a more youthful transcriptional profile.

### 3.7. Autophagy Study

**Figure 7:**
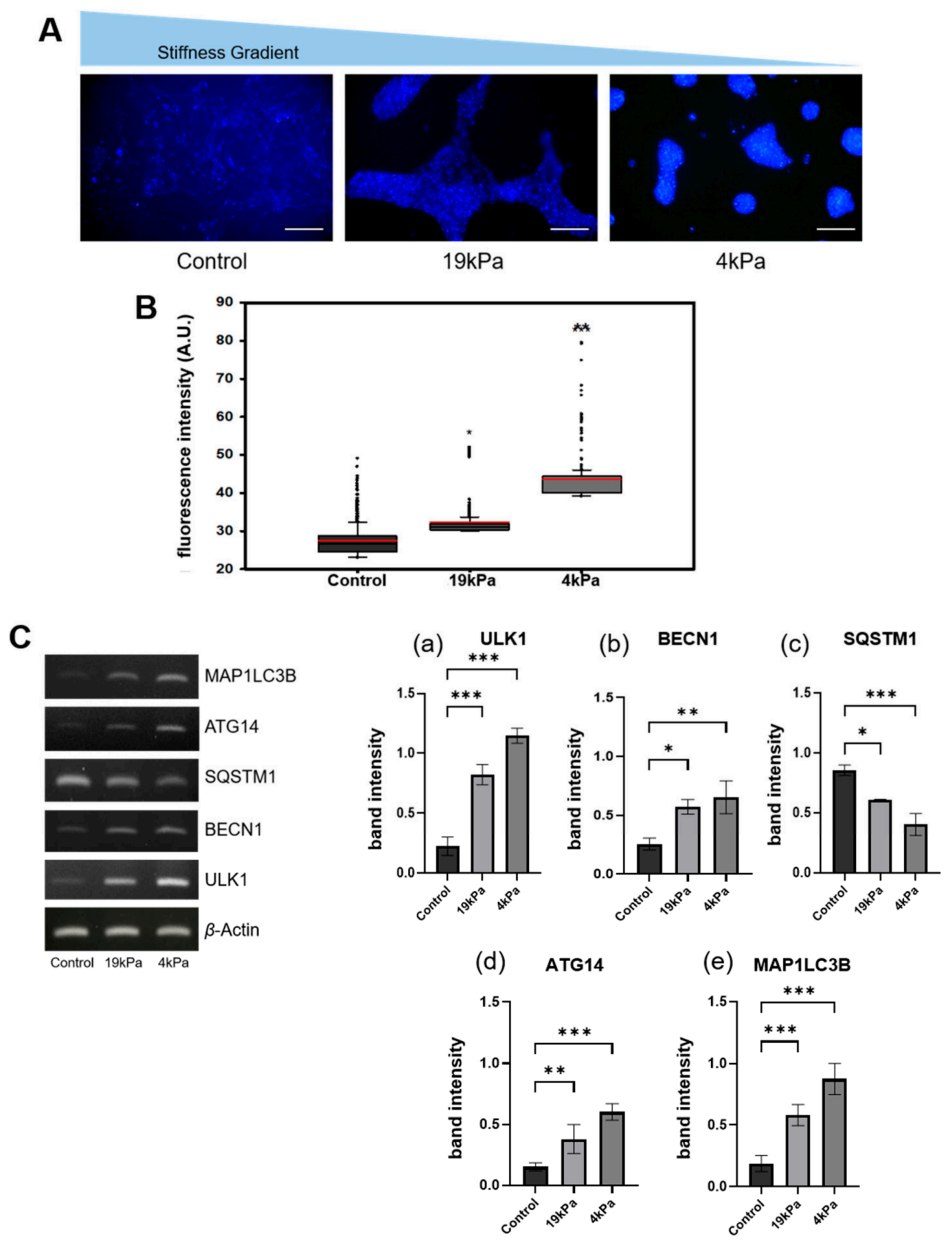
**Autophagy assay.** (**A**) Comparison of autophagy activity in cell cultures on hydrogels of different stiffnesses. Representative fluorescence images show the blue signal labeling autophagic vacuole fluorescence in live cells using a dye that selectively labels autophagic vacuoles. (**B**) Quantification of autophagic vacuole fluorescence, showing the highest expression at 4 kPa and the lowest expression in control. (**C**) RT-PCR analysis showing the effects of mechanical stiffness (Control, 19 kPa, and 4 kPa) on the expression of autophagy-related genes, including (**a**) ULK1, (**b**) BECN1, (**c**) SQSTM1, (**d**) ATG14, and (**e**) MAP1LC3B. Results are expressed as the mean ± SEM from three independent experiments (*n* = 3). Statistical analysis was performed using one-way ANOVA followed by the Holm– Sidak multiple comparisons test (* *p* < 0.05, ** *p* < 0.01, *** *p* < 0.001).

Figure 2 indicates that soft biomechanical conditions exhibit an anti-aging effect compared to hard biomechanical conditions. To elucidate the cellular responses associated with these biomechanical conditions, we specifically analyzed autophagy activity in relation to the mechanical state of the cells. Figure 7A shows a significant increase in autophagy-positive signals under soft conditions compared to hard conditions and the control group. Additionally, the graph in Figure 7B demonstrates that fluorescence intensity increased by 20.1% under the 19 kPa condition compared with the control group, while the 4 kPa condition exhibited a 58.6% increase. Figure 7C evaluates autophagy-related gene expression in the hydrogel culture environment. Compared to the control group, cells cultured under the soft (4 kPa) condition showed that (a) ULK1 expression increased by 411.4%, (b) BECN1 by 155.4%, (c) SQSTM1 decreased by 52.9%, (d) ATG14 increased by 296.2%, and (e) MAP1LC3B expression exhibited a 365.4% increase. These results indicate that a soft mechanical environment significantly enhances autophagic vacuole relative to standard culture conditions.

## 4. Discussion

This study demonstrates that hydrogel stiffness significantly influences cell morphology, proliferation, and aging-associated factors, providing insights into the interplay between biomechanical environments and cellular responses. The findings highlight the relevance of hydrogel mechanical properties in modulating cell behavior, especially in the context of aging and autophagy.

Our observations indicate that hydrogel stiffness directly impacts cellular morphology. Cells cultured on hard hydrogels exhibited a spindle-like, fibroblast morphology with elongated projections, while those on soft hydrogels form spherical, clustered morphologies devoid of extensions. Our data suggest that substrate stiffness not only alters cell shape but may also recapitulate features of cellular aging. These findings indicate that the mechanical environment plays an important role in regulating cytoskeletal architecture. This observation is consistent with previous reports demonstrating that cytoskeletal organization is closely associated with cellular processes such as migration and adhesion [10]. Recent studies have identified actin polymerization as a characteristic feature of brain aging. A Drosophila study further demonstrated that targeting this process can reverse aging-related phenotypes and extend health span [11]. In mammalian systems, solvent-driven rearrangements have also been shown to regulate actin filament assembly and its aging process, providing a basis for the rational development of imaging agents and therapeutic molecules [12]. These studies primarily focus on biochemical and biological targets. Our study adds to this literature by demonstrating that actin organization is modifiable in response to mechanical cues, positioning stiffness-modulated hydrogels as a tool for studying and potentially reversing age-related cytoskeletal phenotypes.

The study also reveals that hydrogel stiffness influences cell proliferation, a critical factor in tissue repair and aging reversal [13]. Cells cultured on soft hydrogels (4 kPa) exhibited the slowest proliferation, while those on stiff hydrogels and standard tissue culture plastic showed higher growth rates. However, reduced proliferation on soft substrates may reflect a transition toward a quiescent or pre-senescent state, potentially favoring longevity [14]. These findings suggest that cellular responses to mechanical cues require an appropriate balance between maintaining proliferative capacity and preventing aging-associated changes. Thus, an optimal range of stiffness may be required to balance cell renewal with maintenance of a youthful phenotype. Our findings suggest that tailoring hydrogel mechanics could help modulate cell aging for regenerative applications. The interpretation is consistent with previous studies suggesting that modulation of hydrogel mechanics may promote regenerative outcomes [15]. Hydrogel stiffness also modulated cellular senescence, as evidenced by stiffness-dependent changes in senescence-associated β-galactosidase (SA-β-Gal) activity. Cells cultured on softer substrates exhibited reduced β-Gal staining, indicating that a compliant mechanical environment suppresses senescence progression. This effect may arise from decreased cytoskeletal tension and reduced nuclear stress, which collectively limit activation of mechanosensitive senescence pathways such as p53/p21 signaling. These findings reinforce the concept that substrate mechanics are key regulators of cellular aging, highlighting the potential of stiffness-tuned hydrogels as experimental platforms to study and modulate senescence-related processes.

The shift in the AMPK-mTOR-AKT signaling triad provides a robust mechanistic explanation for the observed senescence phenotypes. In our model, AMPK activity is inversely coordinated with the mTOR/AKT axis. We observed that its suppression in the control (stiff) condition—driven by the downregulation of its upstream regulator SIRT1—facilitates the sustained activation of mTOR and AKT. This signaling imbalance serves as a central driver of cellular aging, ultimately triggering the transcriptional upregulation of TP53 and CDKN1A, the final effectors of cell cycle arrest [16].

Our integrated analysis further reveals that this process is dictated by a coordinated mechanometabolic signaling axis. In the control (stiff) microenvironment, the activation of the RhoA/ROCK1 pathway was observed, suggesting a “mechanical reset.” The reduction in mechanical strain was accompanied by upregulation of the SIRT1–AMPK axis and inhibition of the p-mTOR/p-Akt pathways, together with suppression of the p53/p21-associated senescence pathway. These findings are consistent with previous reports demonstrating that reduced substrate stiffness promotes metabolic homeostasis and cellular rejuvenation [17,18].

Autophagy is a conserved cellular process that mediates the lysosomal degradation of cytosolic proteins and organelles [19]. Several biomaterials-based studies have explored autophagy for tissue regeneration, considering its high efficacy in anti-aging and aged tissue recovery [20]. In this study, the promotion of autophagy by soft substrates was highly evident, as evidenced by both increased fluorescence intensity and the upregulation of key autophagy-related genes. Specifically, the marked increase in ULK1, BECN1, and ATG14 expression indicates enhanced initiation and autophagosome formation on soft substrates. Furthermore, the increased expression of MAP1LC3B and the decreased expression of SQSTM1 are consistent with enhanced autophagic activity under soft mechanical conditions [21]. Autophagy is a well-known process for maintaining cellular homeostasis and mitigating aging-related stress [22]. This link underscores the potential of using defined biomechanical environments to promote anti-aging effects by restoring cellular clearance mechanisms.

These findings have broad implications for aging research, regenerative medicine, and tissue engineering. While we utilized HEK293 cells as a standardized, highly reproducible human cell model to isolate fundamental mechanotransduction pathways, the downstream pathways identified here, specifically the RhoA/ROCK1-SIRT1-AMPK axis, represent conserved signaling pathways involved in cellular mechanotransduction. However, further validation in diverse cell types and primary aging models is required to confirm the broader physiological relevance of these findings. Future studies should explore intermediate stiffness ranges and combine mechanical cues with biochemical signals to develop more nuanced models of aging. Additionally, incorporating real-time imaging and single-cell transcriptomics may offer deeper insights into the dynamic responses to biomechanical changes. Furthermore, studying long-term effects, immunomodulatory properties, and integration with advanced cellular models can help position mechanically tuned substrates as next-generation tools in cell physiology and aging research.

## 5. Conclusions

This study underscores the pivotal role of the mechanical microenvironment in regulating cell morphology, proliferation, and aging pathways. Soft hydrogels appear to induce a rejuvenated state marked by cytoskeletal reorganization, increased autophagy, and upregulation of longevity-associated genes. In contrast, stiff environments reinforce cytoskeletal tension and growth, mimicking pro-aging conditions. These findings highlight mechanical modulation as a powerful and reversible strategy to guide cellular aging and regeneration, with strong translational potential in tissue engineering and organ-specific therapeutic applications.

## Data Availability

The datasets generated and analyzed during the current study are available from the corresponding authors on reasonable requests.

## Supplementary Materials

The following supporting information can be downloaded at: https://www.mdpi.com/article/10.3390/cells15151380/s1, Figure S1: Original uncropped PCR gel images corresponding to the results presented in Figure 4; Figure S2: Original uncropped Western blot images corresponding to the results presented in Figure 5; Figure S3: Original uncropped PCR gel images corresponding to the results presented in Figure 6; Figure S4: Original uncropped PCR gel images corresponding to the results presented in Figure 7; Table S1: The concentration of acrylamide and bis-acrylamide for the different properties of the hydrogel.
