# Supplementary material for: Reversible Mechano-Regulation of Cellular Senescence: Effects of Substrate Stiffness on Cells
Source: Cells. 2026 Jul 30;15(15):1380. doi: 10.3390/cells15151380 (PMC13464910; doi:10.3390/cells15151380)

Figure S1

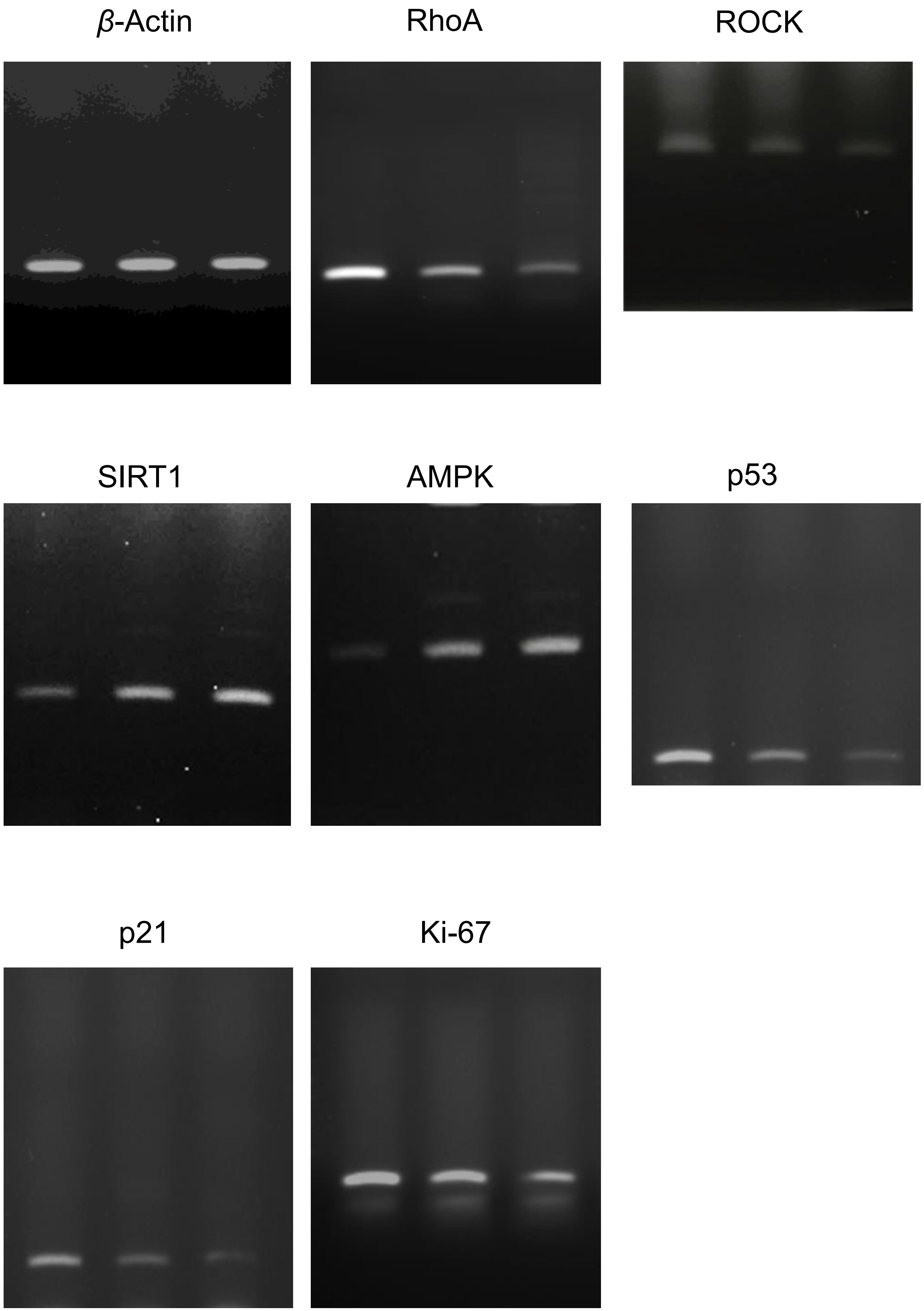

Figure S2

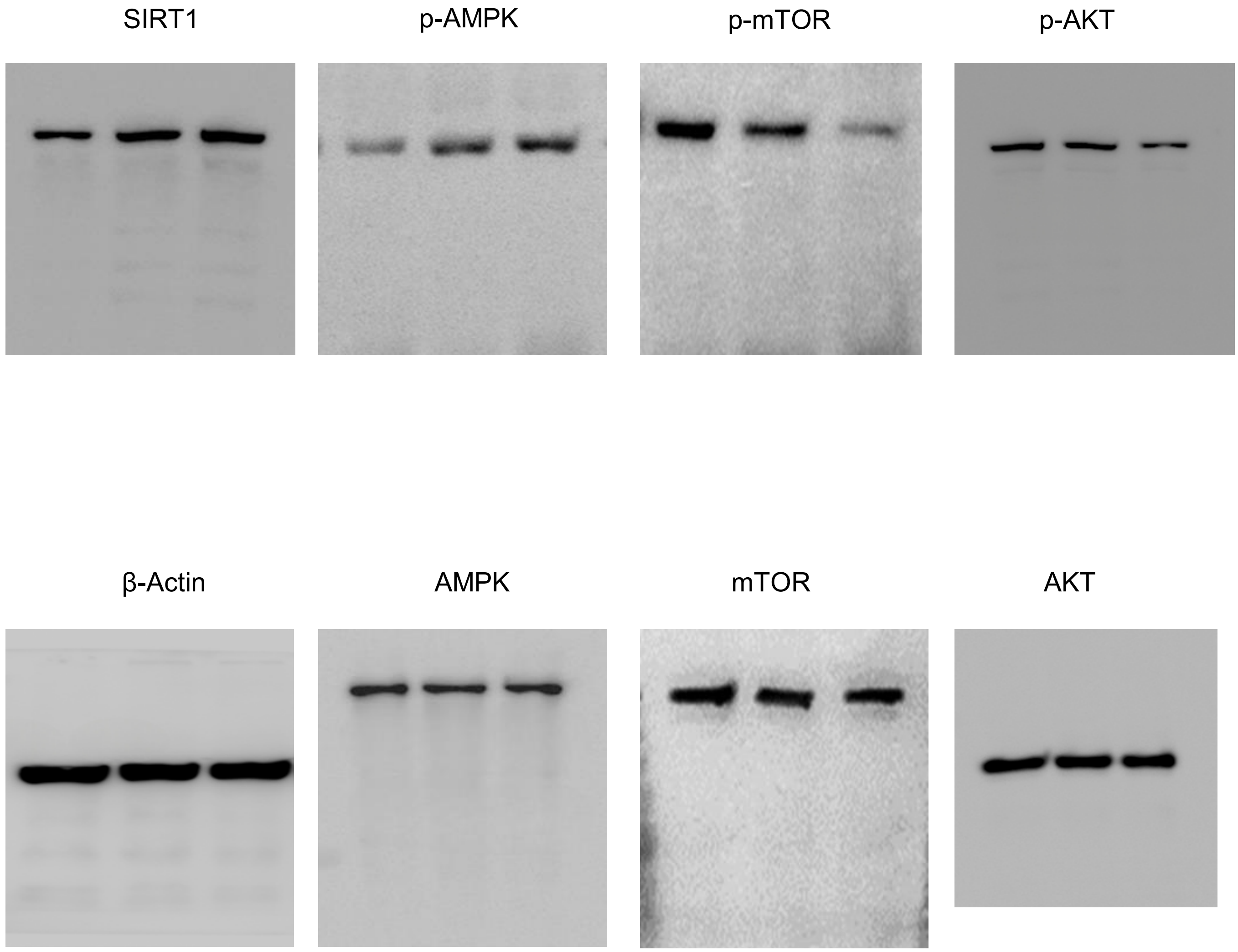

Figure S3

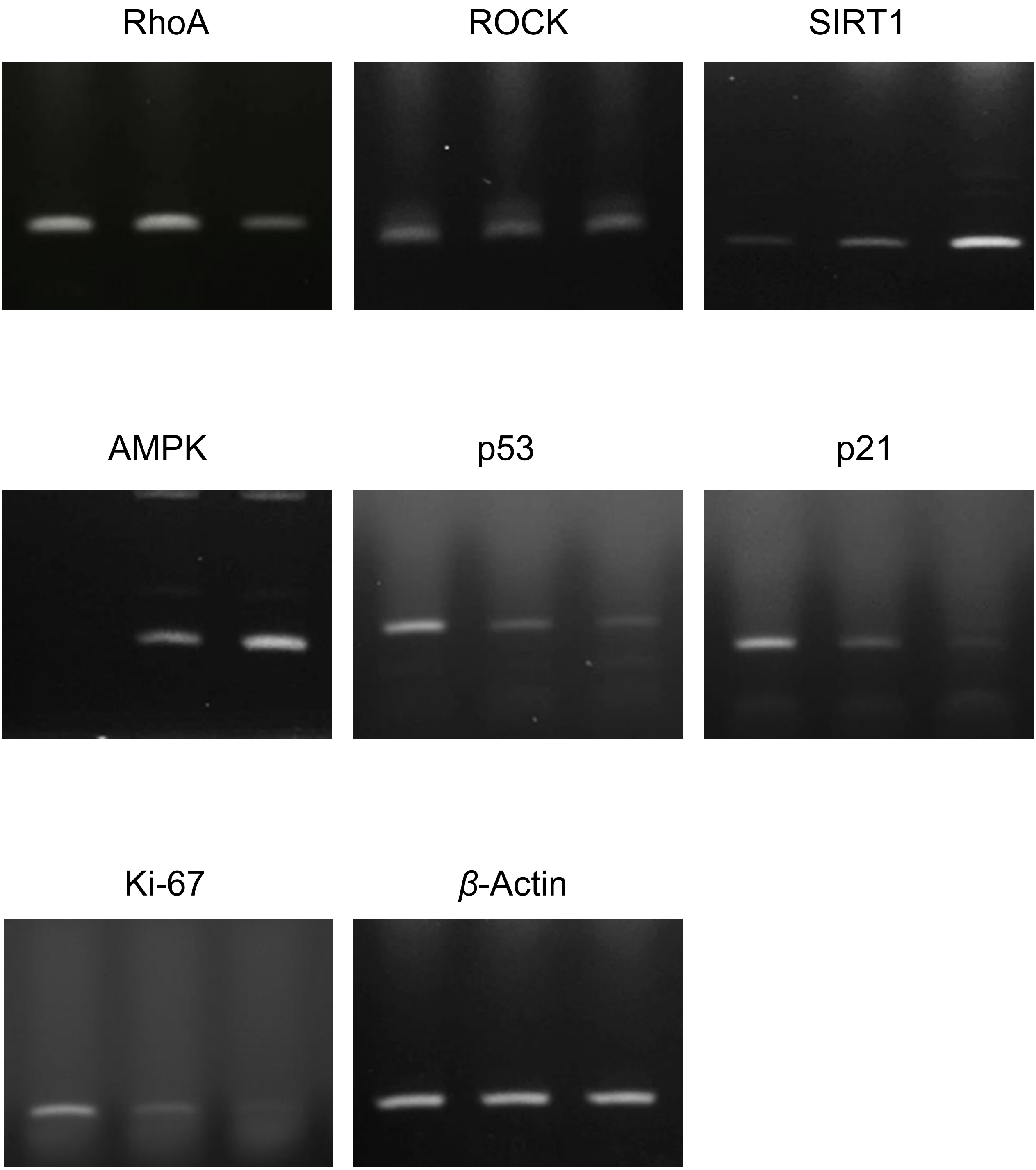

Figure S4

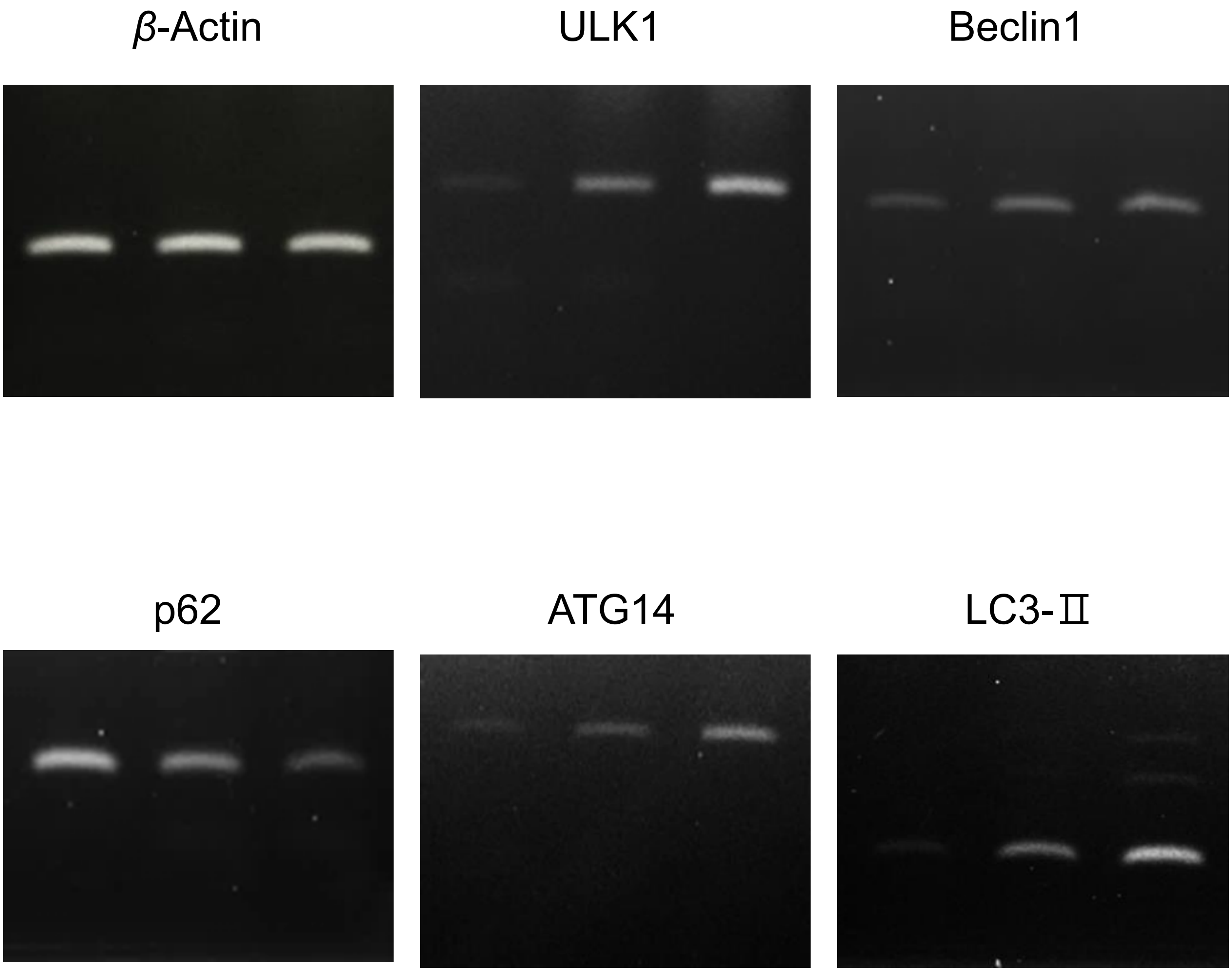

Supplement: Supplementary file 1 [file cells-15-01380-s001.zip › Supplementary Figures.pdf]
